# Hospital Expenditure.—The Commissariat

**Published:** 1895-10-12

**Authors:** 


### The Institutional Workshop.

### HOSPITAL EXPENDITURE.—THE COMMISSARIAT.

### II.—THE COST OF PROVISIONS PER BED.

The difference in cost shown under the head of provisions in hospital accounts is a matter of notoriety. It will be instructive to examine within what limits the figures range, as summarised in *Burdett's Hospital and Charities Annual*, 1895, and to consider the full bearing of these figures on practical housekeeping.

In the uniform system of accounts nine items are specified under the head of provisions, as follows: 1, meat; 2, fish, poultry, &c.; 3, butter, cheese, &c.; 4, eggs; 5, milk; 6, bread, flour, &c.; 7, grocery; 8, vegetables; 9, malt liquors. In the Annual the cost of malt liquor is deducted from the total and classed under the head of alcohol, but the amount spent on this item is not such as to affect the averages to any appreciable degree. In a few hospitals the provision accounts for patients, nurses, and doctors are stated separately, but this is the exception. In every case the total represents the cost of all the food consumed in the institution divided by the daily average of occupied beds.

The following hospitals, arranged in order of cost, have a daily average of over 100 occupied beds:—

Average Cost of Provisions per Occupied Bed.

| Per Year. £ | | Per Week. s. | d. | | |
|---|---|---|---|---|---|
| 28½ | ... | 10 | 11 | ... | St. Mary's. |
| 27½ | ... | 10 | 6¾ | ... | London. |
| 27¼ | ... | 10 | 5½ | ... | St. Thomas's. |
| 26¾ | ... | 10 | 3½ | ... | St. Bartholomew's; Royal Free. |
| 25¾ | ... | 9 | 11 | ... | King's. |
| 25½ | ... | 9 | 9½ | ... | Charing Cross; German. |
| 25¼ | ... | 9 | 8¼ | ... | St. George's. |
| 24¾ | ... | 9 | 6½ | ... | Guy's. |
| 24½ | ... | 9 | 5 | ... | Sheffield General. |
| 24¼ | ... | 9 | 3½ | ... | Seamen's Hospital. |
| 24 | ... | 9 | 2½ | ... | Westminster. |
| 23¾ | ... | 9 | 1½ | ... | Sussex County. |
| 23½ | ... | 9 | 0¼ | ... | Radcliffe, Oxford. |
| 22½ | ... | 8 | 7½ | ... | North Staffordshire Infirmary. |
| 22 | ... | 8 | 5¼ | ... | Adelaide Hospital, Dublin. |
| 21½ | ... | 8 | 3¼ | ... | Nottingham General. |
| 21¼ | ... | 8 | 2 | ... | Glasgow Western Infirmary. |
| 21 | ... | 8 | 0¾ | ... | South Devon and East Cornwall. |
| 20¾ | ... | 7 | 11¾ | ... | Birmingham General. |
| 20½ | ... | 7 | 10½ | ... | Edinburgh Royal Infirmary.<br>Liverpool Northern. |
| 20 | ... | 7 | 8 | ... | York County; Norfolk and Norwich. |
| 19¾ | ... | 7 | 7¼ | ... | Glasgow Royal Infirmary. |
| 19½ | ... | 7 | 6 | ... | Manchester Royal Infirmary. |
| 19¼ | ... | 7 | 4½ | ... | Newcastle Royal Infirmary. |
| 19 | ... | 7 | 3½ | ... | Birmingham Queen's; Preston Roy. Infiry. |
| 18¾ | ... | 7 | 2½ | ... | Bristol Royal Infirmary; Chester General. |
| 18½ | ... | 7 | 1¼ | ... | University; Leicester Infiry.; Liverpool Royal Infiry.; Liverpool Roy. Southern. |
| 18¼ | ... | 6 | 11¾ | ... | Devon and Exeter. |
| 18 | ... | 6 | 10¾ | ... | Leeds General Infirmary; Dundee Royal Infirmary. |
| 17¾ | ... | 6 | 10 | ... | Bristol General; Royal Portsmouth. |
| 17½ | ... | 6 | 8½ | ... | Hull Royal Infirmary. |
| 17¼ | ... | 6 | 7¼ | ... | Bradford Infirmary; Sunderland Infiry. |
| 17 | ... | 6 | 6¼ | ... | Wolverhampton and Staffordshire; Aberdeen Royal Infirmary. |
| 16¾ | ... | 6 | 5¼ | ... | Belfast Royal. |
| 16½ | ... | 6 | 4¼ | ... | Royal Berkshire. |
| 15¾ | ... | 6 | 0¾ | ... | Paisley Infirmary. |

It will be seen that nothing could be finer than these shades of difference, gliding down by a few yearly shillings, and reaching a gross total between the two extremes of no more than £12. It would be hard, indeed, to pause in passing either up or down this gently sliding scale and pronounce at what point economy ceased or parsimony began. An examination of the weekly column will show even more clearly the minuteness of these variations in cost.

The smaller hospitals present stronger contrasts, although the majority are to be found walking safely in the *via media*. The following are hospitals with a daily average of between thirty and one hundred occupied beds:—

Average Cost of Provisions per Occupied Bed.

| Per Year. £ | | Per Week. s. | d. | | |
|---|---|---|---|---|---|
| 47¾ | ... | 18 | 4½ | ... | Metropolitan. |
| 36¾ | ... | 14 | 1½ | ... | North-West London. |
| 34 | ... | 12 | 0½ | ... | Durham County. |
| 29¾ | ... | 11 | 5 | ... | Stockport Infirmary. |
| 27¾ | ... | 10 | 5¼ | ... | Cheltenham General. |
| 26¾ | ... | 10 | 3½ | ... | Macclesfield General. |
| 26½ | ... | 10 | 1 | ... | Blackburn. |
| 26 | ... | 9 | 11 | ... | Hartlepool. |
| 24½ | ... | 9 | 5 | ... | Walsall Cottage. |
| 24 | ... | 9 | 2½ | ... | Suffolk General. |
| 23¾ | ... | 9 | 1½ | ... | Royal Surrey County. |
| 22¼ | ... | 8 | 7¾ | ... | Perth County; Bolton Infirmary. |
| 22¼ | ... | 8 | 6¼ | ... | Coventry and Warwick. |
| 21½ | ... | 8 | 3¼ | ... | French Hospital. |
| 21¼ | ... | 8 | 2 | ... | Salop Infirmary. |
| 20¾ | ... | 7 | 11¾ | ... | Warneford, Leamington, &c.<br>Stockton and Thornaby. |
| 20½ | ... | 7 | 10½ | ... | Great Northern Central.<br>Ayr County. |
| 20¼ | ... | 7 | 9 | ... | Salford Royal. |
| 19¾ | ... | 7 | 7 | ... | Taunton and Somerset. |
| 19 | ... | 7 | 3½ | ... | S. Charitable Infirmary, Cork.<br>Gloucester General. |
| 18¾ | ... | 7 | 2½ | ... | Derbyshire R. Infirmary; Halifax Infirmary; Staffordshire Gen.; E. Suffolk. |
| 18¼ | ... | 6 | 11¾ | ... | Hants Royal County; Dumfries Royal. |
| 18 | ... | 6 | 10¾ | ... | Salisbury; Kilmarnock. |
| 17¾ | ... | 6 | 10 | ... | West London; Swansea General. |
| 17½ | ... | 6 | 8½ | ... | Essex and Colchester. |
| 17¼ | ... | 6 | 7¼ | ... | Worcester General. |
| 17 | ... | 6 | 6¼ | ... | Hereford General.<br>Lincoln County.<br>Royal Albert. |
| 16¼ | ... | 6 | 2¾ | ... | Sheffield Public. |
| 15¾ | ... | 6 | 0¾ | ... | Queen's County. |
| 15½ | ... | 5 | 11 | ... | Bootle Borough. |
| 14½ | ... | 5 | 6¼ | ... | Fermanagh County. |
| 12¼ | ... | 4 | 8¼ | ... | Down County. |

Here we begin at £47 15s. and end at £12 5s., with a difference between these extremes of £35 10s. And it is noteworthy that the number of occupied beds in the Metropolitan and County Down Infirmary is about the same. It is such abnormal examples as these which render the averages of the hospitals, London or provincial, taken in groups, of no avail for comparison. One ill-managed hospital will throw out the average for a dozen others, and render all conclusions based on the figures abortive.

It is not our purpose to consider at any length the management of the special hospitals, nearly all of which present distinct features, controlling their expenditure. But it may be pointed out that between the highest and lowest of these institutions the difference is startling indeed, the extreme at one end being the British Lying-in Hospital, expending £59 per year for provisions on each occupied bed; and at the other, the Derbyshire Hospital for Sick Children, spending £7 10s.; or for adults, the National Orthopædic Hospital, standing at £10 15s.

The tables given do not mark a finer gradation than a yearly difference of 5s. per bed, or considerably less than one farthing a day. It would be extremely easy for most housekeepers to spend an extra farthing daily on each person's food in the household, without producing any very startling novelties of diet, or being very sure what became of the money. But an extra farthing (or less) on each person's diet daily does not by any means fairly state the case for the hospital. Grant it to be a symptom of extravagance. It embraces then the sum total of lavishness, not merely in the patient's diet (though an extra crust of bread or draught of milk would suffice in his case), but in the diet of his share of the nurse who attends to him, of the servant who cleans the ward, cooks his dinner, and admits his friends; of the doctor who probes his wound, and of the secretary who calculates his expenses. A fraction of a farthing a day is not a great discrepancy when these little matters are duly weighed. Nor, perhaps, would eightpence a day, which is the extreme difference in cost of provisions among the larger hospitals, be a difficult sum to spend on each additional bed without conscious mismanagement. Yet, among these fractions lie manifold gradations between waste and parsimony, and the neglected farthings lie hidden somewhere at the root of half the desperate deficits and hampered finances under stress of which the average hospital performs its work.

